# Supplementary material for: Virome Profiling of an Eastern Roe Deer Reveals Spillover of Viruses from Domestic Animals to Wildlife
Source: Pathogens. 2023 Jan 18;12(2):156. doi: 10.3390/pathogens12020156 (PMC9959412; doi:10.3390/pathogens12020156)
Supplement: Supplementary file 1 [file pathogens-12-00156-s001.zip › pathogens-2155006-supplementary.pdf]

Table S1. Primer information used in this study.

Application	Primer name	Sequence(5'-3')	Target
Species identification	COI-TF	5'GGTCAACAAATCATAAAGATATTGG 3'	COI
	COE-TR	5'TAAACTTCAGGGTGACCAAAAAATCA 3'	
PCR detection	KoV-JC-1F	5'TGTTGCTTCCTCCACCAACC 3'	KoV
	KoV-JC-1R	5'CAGTGCGAGGCAATCGTC 3'	
	BoV-NSVP-F	5'CAGGTGTAAAGCGTCGCCTGGAC 3'	BoV
	BoV-NSVP-R	5'GCCTCCAGCATCTCAGCGTCACTT 3'	
	XKC1-F	5'CAAGTTCAAGCTCGACGCAG 3'	GmV-CpXKC1
	XKC1-R	5'CTTGAACTTGCGCATGCTGA 3'	
Gap-filling	BoV-NSVP-F	5'CAGGTGTAAAGCGTCGCCTGGAC 3'	BoV
	BoV-NSVP-R	5'GCCTCCAGCATCTCAGCGTCACTT 3'	
	BoV-VP2-F	5'CACACAAAGCACGTGGTCCGAT 3'	
	BoV-VP2-R	5'CACACAAAGCACGTGGTCCGAT 3'	
	BoV-NS1-F1	5'ATGAATTKTGGGCGGGCTTA 3'	
	BoV-NS1-R1	5'AACGGAGTTGCTCTGTCAGG 3'	
	BoV-NS1-F2	5'CTCTGTCTGYCAGAAGGCCG 3'	
	BoV-NS1-R2	5'GCCTGCTCTACCCAATCGTT 3'	
	BoV-NS1-F3	5'CCGCAGACTCCTGTTGTGAT 3'	
	BoV-NS1-R3	5'GCTGCCACTGAGCGTAAAAC 3'	
	BoV-gF1	5'CATTGGCTGCGTCTACCAG 3'	
	BoV-gR1	5'TCACACATCCGTACAGC 3'	
	BoV-gF2	5'GCTGTACGGATGTGTGA 3'	
	BoV-gR2	5'CTGAAGCTGCCACTGAGCG 3'	
	BoV-gF3	5'CGTGAGAGGCATAGACTTAG 3'	
	BoV-gR3	5'TCTTAAAGTTACAGTACCCTGC 3'	
	BoV-gF4	5'CTCGCGGATACAAAGGGCAA 3'	KoV
	BoV-gR4	5'GTCTGCCTGTGGAATGGCGTA 3'	
	Kov-gF	5'CTCGCCAGCGACGCGATYAAAT 3'	
	Kov-gR	5'CAGTCTGGATACGCCTGCACGA 3'	
